# [Corrigendum] Silencing glioma-associated oncogene homolog 1 suppresses the migration and invasion of hepatocellular carcinoma *in vitro*

**DOI:** 10.3892/ol.2026.15596

**Published:** 2026-04-15

**Authors:** Zeming Hu, Fangfang Xie, Ang Hu, Mengjing Xu, Yuwen Liu, Jiankang Zhang, Jianbo Xiao, Yunlei Song, Jianing Zhong, Bin Chen

Oncol Lett 20: 228, 2020; DOI: 10.3892/ol.2020.12091

Following the publication of the above article, an interested reader has drawn to the authors’ attention that, in the originally published version of Fig. 3 on p. 4 showing the results of cell invasion assay experiments, the ‘CTRL’ and ‘NC’ data panels, and the si-Gli1/50 nM and si-Gli1/100 nM data panels, respectively showed overlapping sections of data, such that data which were intended to show the results of differently performed experiments had been derived from a pair of original sources. In addition, the authors themselves have noted that several funding projects were inadvertently included in the Funding section of the Declarations that were not related to this study. The following grants were incorrectly listed, and should be removed from the funding section: (1): The National Natural Science Foundation of China (grant no. 81760160); (2) The Natural Science Foundation of Jiangxi Province (grant no. 20151BAB205044); and (3) The Science and Technology Research Project of Jiangxi Provincial Education Department (grant no. 180797). Therefore, the Funding section for this paper ought to have read simply as follows: “The present study was funded by grants from the Provincial Innovation Training Project of Jiangxi Provincial Undergraduates (grant no. S202010413009) and the Science and Technology Innovation Project for Undergraduates of Gannan Medical University (grant no. BKSZR201902).”

After having consulting their original data for Fig. 3, the authors have realized that this figure was inadvertently assembled incorrectly; specifically, in Fig. 3C, the image for the ‘NC’ group was inadvertently replaced with the image for the ‘CTRL’ group, and the image for the ‘si-Gli1/50 nM’ group was inadvertently replaced with the image for the “si-Gli1/100 nM” group. The revised version of Fig. 3, now showing the correct data for the ‘NC’ and the ‘si-Gli1/50 nM’ data panels, is shown on the next page. The authors regret the errors that were made in assembling this figure, and thank the Editor of *Oncology Letters* for granting them the opportunity to publish this Corrigendum. All the authors agree with the publication of this Corrigendum, and apologize to the readership for any inconvenience caused.

## Figures and Tables

**Figure 3.**
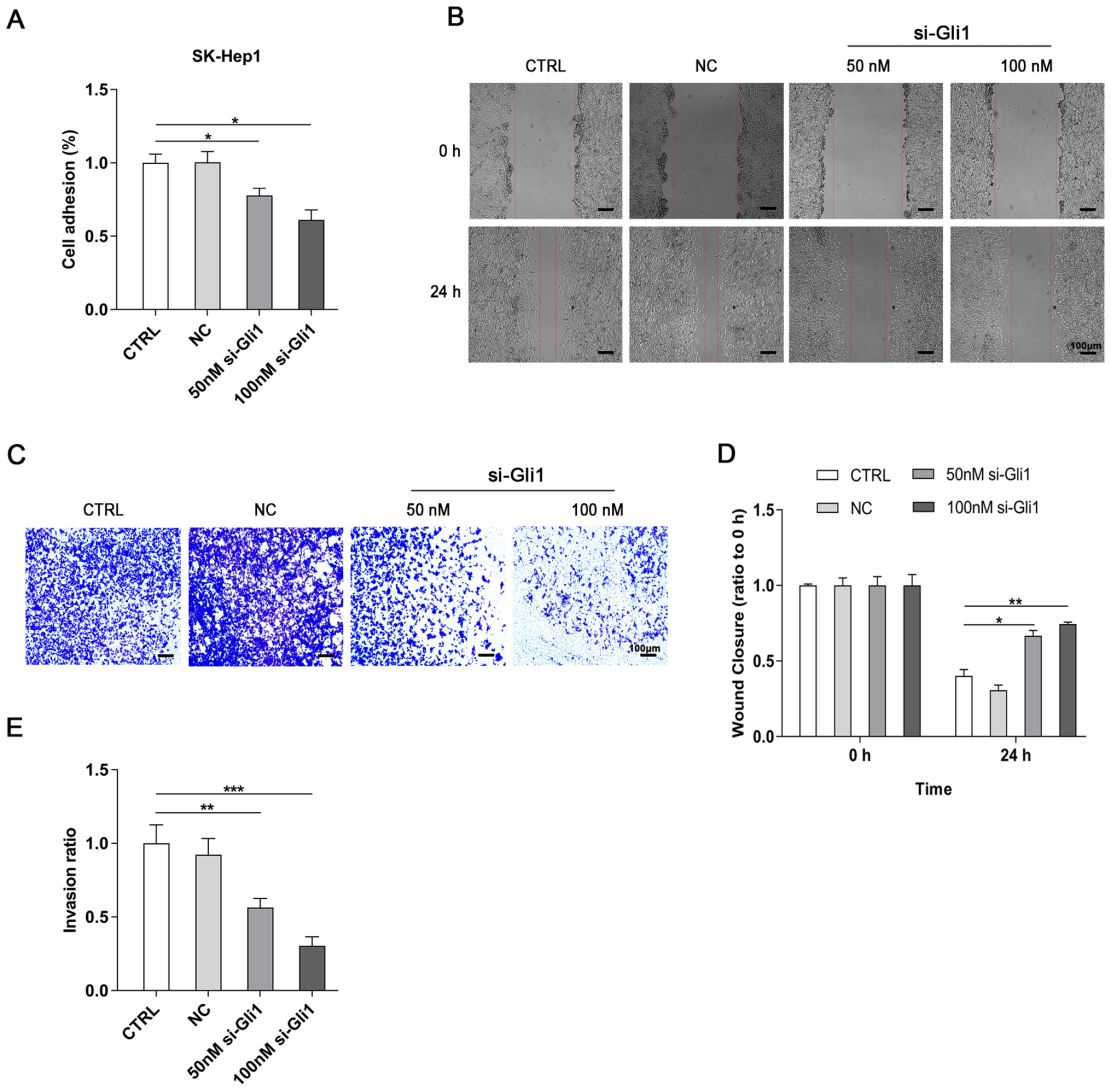
Gli1 interference inhibits cell adhesion, migration and invasion in SK-Hep1 cells. (A) Cell adhesive ability of hepatoma cells was significantly suppressed in the si-Gli1 groups compared with the CTRL group using cell adhesion assay. (B and C) Images and (D and E) histograms showing that Gli1 siRNA markedly decreased the migration and invasion abilities of SK-Hep1 cells as demonstrated by the wound healing and Matrigel invasion assays, respectively. Scale bar, 100 µm. *P<0.05, **P<0.01 and ***P<0.001 vs. CTRL group. Gil1, glioma-associated oncogene homolog 1; si, small interference; CTRL, blank control group; NC, negative control siRNA group.

